# Medicine

**Published:** 1892-09

**Authors:** R. Shingleton Smith


### Progress of the Medical Sciences.

---

### MEDICINE.

"The treatment of pneumonia has long been a famous battle ground whereon physicians have met in severe combat in the attempt to prove the special advantages of varying methods of therapeutics. I myself was brought up in the camp of a famous general (the late Professor Hughes Bennett, of Edinburgh), who took the field with overweening confidence, and with supporting and restorative treatment declared that he won all along the line over his opponents who ventured to employ such drugs as antimony and mercury, or were so ignorant as to bleed in this disease. This battle is now fought out, and in the light of modern therapeutics we find that the truth was really on neither side."[1]

That the heart is the chief source of danger in pneumonia all are agreed, and on this subject Dr. J. M. Anders[2] read a paper at the meeting of the American Medical Association at Detroit, in which he said that the causes of cardiac weakness are threefold. We may have an absorption of toxic ptomaines as a result of the disease-process, with a weakening of the heart-muscle. There may be pulmonary obstruction, produced by the presence of the exudate acting as a local barrier to the integrity of the circulation. Finally, we may have the formation of cardiac thrombi, which are found in the right ventricle: the latter becomes dilated, and thus there is failure of heart-power.

The signs of muscular degeneration appear as a feebleness and shortening of the first apex-sound, and a similar condition of the aortic second sound. The pulmonary second sound may be accentuated, at least temporarily.

Hence treatment by digitalis has an ardent advocate in Petresco,[3] whose doses are enormous, amounting to 90

[1] Sir Dyce Duckworth, *Internat. Clinics*, vol. iv., p. 93.
[2] *Med. Rec.*, July 2, 1892, p. 21.
[3] *Annual Univ. Med. Sci.*, 1892, vol. v., A—60.

grains of the leaves in 24 hours; these doses were well borne, and no single case of poisoning had occurred. Fikl[1] has also reported very brilliant results from a similar method, and Hershey[2] gives a hot infusion of digitalis in tablespoonful doses every hour, together with 10 grains of calomel at the beginning. Balfour[3] has also warmly advocated digitalis in combination with chloral. The dose must vary with the age of the patient: for adults, 20 grains of chloral dissolved in a half-ounce of infusion of digitalis, and subsequently half of this dose every hour, until the temperature falls to normal; and this, according to Balfour, it rarely fails to do. Other good clinical observers advocate what are commonly considered to be heroic and dangerous doses of this potent and useful drug: from time to time large doses have been given, as for instance in the treatment of delirium tremens, where I have frequently seen excellent results from half-ounce doses of the tincture; but the profession generally has not accepted the statements made as to the safety of these heroic doses.

In the face of the accumulating evidence of numerous good observers and of the fact that the restorative treatment of pneumonia, as judged by the results, has been a disastrous failure, it becomes a question whether there is not more safety in heroic doses of a dangerous drug than in the unchecked processes of an exceedingly fatal disease.

In a paper on "The Teachings of Experience and of Rational Therapeutics in the Treatment of Pneumonia,"[4] Dr. Boardman Reed quotes much favourable testimony as to the abortive power of veratrum viride in the first stage of sthenic cases of this disease. His own experience corroborates this, and also leads him to consider that the drug in proportionate doses is of great service even in asthenic cases. Dr. Reed's plan of treatment is to place the patient in a well-ventilated room having a temperature of 65° to 70°. He allows as much cold water as the patient desires, and at first just as much simple bland nourishment as the appetite demands. His usual prescription for a pneumonia seen early is somewhat as follows:—

℞ Tr. Verat., Vir. ♏xvi.

Vin. Antim. ʒj.

Tr. Opii. Deod. ♏xx. to xxx.

Liq. Ammon. Acet. q.s. ad ℥iv. M.

Sig. Two teaspoonfuls in a draught of water every two hours, day and night.

[1] *Annual Univ. Med. Sci.*, vol. i., A—7.
[2] *Med. News*, vol. lix., 1891, p. 65. [3] *Ed. Med. Journ.*, Nov., 1891.
[4] *Ther. Gaz.*, Mar., Apr., 1892.

Two grains of quinine are usually given in addition three or four times a day. Dr. Reed claims that nearly all cases which he has recently had to treat have thus been terminated in the first stage, and he asserts that general experience has abundantly proved that acute primary pneumonia can to this extent be aborted by a plan of treatment, including, internally, frequently repeated moderate doses of veratrum viride, combined with adjuvants, which dilate the capillaries of the skin and body generally, at the same time that they stimulate the kidneys and support the heart; and externally, either such hydro-therapeutic measures as will most conveniently meet the threefold indication of lowering the temperature, diverting blood from the inflamed area, and stimulating the heart and respiratory centre, or other local energetic measures by which the same results can usually be accomplished less promptly and certainly, but also with less opposition from the friends of the patient. Dr. Reed adds that when the plan has been instituted too late to abort the disease, it has proved, even then, the most curative of any of which extensive trials have been made.

Amongst the modern curiosities in therapeutics is the treatment of acute pneumonia by artificial abscess.[1] Professor Fochier, in the French Academy of Medicine on April 26th, read a long paper on the production of determinant abscess by means of subcutaneous injections of essence of turpentine into the deltoid or hypogastric regions. Cases are given by Bard and Dieulafoy, in which the results of the method were satisfactory.

As regards the genesis of pneumonia, Dr. W. W. Pennell[2] discusses the question whether pneumonia is a general fever or a local disease. Bacteria, he believes, play only a secondary part. The inflammation produced experimentally by pure cultures differs from that of the primary cultures. There is not an exact analogy with the so-called specific diseases. The infectious character of the disease is not observed in country districts.

As against the specific character are the following arguments:

(1) More than one germ is found. (2) Patients are liable to subsequent attacks. (3) It is not contagious. (4) There are no prodromata. (5) There are no sequelæ. (6) There is a distinct crisis unless peri-infiltration or abscess result. (7) Antiphlogistic treatment will often abort the disease.

[1] *Practitioner*, July, 1892, p. 51.
[2] *Med. Rec.*, July 2, 1892.

The essential factors in the etiology of the disease are believed to be threefold:

(1) Predisposition. (2) A loss or lack of normal tissue resistance. (3) An exciting cause.

On the other hand, Emmerich[1] claims that an outbreak of pneumonia may be prevented by protective injections; immunity is to be obtained by injections of blood or juice from the tissues of immunized animals. Herzog,[2] who has studied the experiments of Emmerich and observed his results, considers them very favourable; and they seem to him to warrant the belief that the juice of the immunized rabbits as prepared by Emmerich will not only confer immunity, but also obtain cures.

Little advance has been made in the question which a little more than a year ago eclipsed all else; that is, the value of tuberculin. The only noteworthy experiments are those of Klebs,[3] and Hunter in conjunction with Watson Cheyne.[4] The chief danger attaching to the crude tuberculin appears to be due to a by-product acting on the heart and producing a profound fall of blood pressure. Koch for a time had failed to purify the preparation from these products; but Klebs has succeeded in obtaining a purified product, called tuberculocidin, from which the by-effects on the heart are absent. Dr. Carl Sprengler,[5] of Davos, undertook a confirmatory testing of this purified preparation. He believed that the entire absence of irritant properties is not altogether an advantage in phthisis. He found that the drug removed dyspnœa and reduced hectic, and modified cases of active phthisis by eliminating the worst features; but "tuberculocidin lacks the necessary activity." Koch aims at reaction, alteration, and destruction; Klebs at preservation and resolution; whilst Sprengler maintains that the reconciliation of these views is unconditionally necessary to the perfecting of the method of treatment: hence he advises a tuberculin-tuberculocidin mixture.

Of the numerous other methods of treatment of phthisis, creasote and guaiacol still take the lead, and evidence of their unquestionable utility accumulates rapidly. Dr. Geo. H. Penrose, of Washington, remarks that they are of infinite worth, and are more than palliative in their action. The principal question is one of dose: some observers, like Som-

[1] *Annual Univ. Med. Sci.*, 1892, vol. i., A—3. [2] *Ibid.*
[3] *Therapist*, April, June, 1892. [4] *Lancet*, 1891, vol. ii., p. 349.
[5] *Therapist*, June, 1892.

merbrodt, content themselves with moderate doses; others give these drugs in increasing amount till tolerance is established and fabulous quantities are taken.

The following case is reported by Dr. Freudenthal.[1]

Woman, æt. 30. Advanced phthisis, both lungs. Feb. 24, 1891.

Apl. 15. Taking 60 drops of creasote daily. (60 of mixture [2 parts, Tinct. Gent. Co., 1 part, Creasote] 3 times daily.)

July 5. Taking 100 drops of creasote daily. Dizzy at times after dose.

Dec. 6. Taking 200 drops of mixture twice daily.

Jan., 1892. Took 300 drops twice daily=100 drops of creasote. (By measure, 5 grammes of creasote daily.)

Jan. 29. Took same dose; went for walk; did not feel well, came home and drank a glass of wine. Feeling weak, took another dose of 300 drops of mixture; was unconscious for eight to nine hours, narcotised, stertorous, eyes closed, *râles* over whole chest, trismus. Lips cyanotic, pupils contracted. P. 128; R. 30. Paralysis of all reflex movement; urination in bed, no carbolic staining of clothing. No evil results followed. No damage to kidneys.

Symptoms: Those of carbolic acid poisoning.

Afterwards dose increased to 500 drops twice daily. Tolerance soon established.

Opposed to the practice of giving large doses is the experience of Dr. Kinnicutt,[2] whose conclusions are, that both creasote and guaiacol in certain forms can be given in very large doses with entire tolerance and without injurious effects; that such dosage apparently possesses no advantages over a much smaller one, and that it has no greater effect upon hectic and night sweats; that subcutaneous injections of the drug possess no advantages over administration by the mouth; that whatever beneficial influence creasote may exert in pulmonary tuberculosis can be effected with a comparatively small dosage, and that favourable results can be expected only by its continuous and prolonged employment.

The treatment of hæmoptysis by drugs is a subject on which the faith of the profession was much shaken by the

[1] *Med. Rec.*, April 23, 1892. [2] *Ibid*, May 21, 1892.

Harveian orator of 1890, Dr. Andrew, whose criticisms on the methods commonly in vogue have left us with no trustworthy guide in some of the most critical cases we are ever called upon to conduct. Dr. Vincent D. Harris has presented the subject in an excellent paper,[1] in which he comments on the use and abuse of venesection and emetics in cases of profuse bleeding when it is necessary to clear the air-passages of blood. He also mentions the utility of morphia injections, which I believe to be by far the most beneficial remedy, inasmuch as the morphia stops the cough and favours mechanical rest to the bleeding point.

Of the drugs which act by increasing the tendency of the blood to clot at the seat of hemorrhage, the chloride of calcium, as suggested by Wright,[2] is worthy of a good trial. It may easily be given in drachm doses of the liquor calcis chloridi every hour, and a small dose of morphia with each dose has given excellent results in two cases in which it has been recently tried.

The researches of Sidney Martin in diphtheria have been very ably summed up by Dr. Woodhead in his address on Bacteriology at the British Medical Association meeting in Nottingham, and form an excellent sequel to a comment on this subject in the *Journal* a year ago. It was then pointed out that the Klebs-Loeffler bacillus was the primary local infective agent, and that a secondary infection by the products of bacillary activity was necessary to the manifestation of any constitutional symptoms. Martin has succeeded in separating definite substances, each of which, appearing to exert a definite effect on certain tissues and functions, gives rise to special symptoms and pathological changes. "Bacteriological chemistry thus supplies another diagnostic factor in the detection of specific infective disorders. Martin points out that to Koch's four cardinal rules a fifth may be added; that is, that the secondary infective agent—the chemical poison separable from the tissues in the natural disease—should exert a definite physiological action, and that this should be similar, both chemically and physiologically, to the products obtained from a pure artificial culture of the primary infective agent—the micro-organism." Attempts have been made by Roux and Yersin to devise a method which may be of use in clinical diagnosis, and they maintain[3]

[1] *St. Bartholomew's Hospital Reports*, vol. xxvii., 1891.
[2] *Brit. Med. Journ.*, 1891, vol. ii., p. 1306.
[3] *Ibid.*, Aug. 6, 1892, p. 287.

that it is possible for all those engaged in attendance on patients in whom the presence of diphtheria is suspected, to settle the question for themselves by an experimental inoculation of blood serum. The appearance and rapidity of the growth alone are quite sufficient to justify the medical attendant in deciding that he is dealing with diphtheria. Beyond this point the investigation may be taken up by the expert, if the more elaborate chemical examinations are not within the powers of the practitioner himself.

R. SHINGLETON SMITH.

